# Neuroprotective and antioxidant activities of different polarity parts of the extracts of the *Ginkgo biloba* leaf and *Zingiber officinale* rhizome from Yongzhou

**DOI:** 10.3389/fchem.2022.984495

**Published:** 2022-09-07

**Authors:** Zuoying Huang, Tingting Yuan, Jiayi Chen, Mihan Jiang, Rongling Yan, Wencai Yang, Liqian Wang, Yang Liao, Guowen Huang

**Affiliations:** ^1^ College of Life Sciences and Chemistry Engineering, Hunan University of Science and Engineering, Yongzhou, Hunan, China; ^2^ Key Laboratory of Comprehensive Utilization of Dominant Plant Resources in Southern Hunan, Yongzhou, Hunan, China; ^3^ Hunan Provincial Engineering Research Center for Ginkgo Biloba, Yongzhou, Hunan, China

**Keywords:** neuroprotective activity, antioxidant activity, correlation, *Ginkgo biloba*, *Zingiber officinale*, blood physiology

## Abstract

In order to make better use of the two local dominant plant resources of *Ginkgo biloba* and *Zingiber officinale* from Yongzhou in Hunan province, the *in vitro* neuroprotective and antioxidant activities of extracts from the *G. biloba* leaf and *Z. officinale* rhizome, and the correlation between these two kinds of activities, were analyzed. The *in vivo* effects of these two plant extracts on aged mice blood physiology and central neuron cell activity were then determined after continuous gavage with the best polarity part at different concentrations (2, 4, 8 mg/ml). The results showed that the cell survival rate and superoxide dismutase (SOD) activity of the induced injury central neurons increased, although the malondialdehyde (MDA) content decreased gradually with the extract concentrations increasing in a certain range. Different polarity parts performed differently, even though they had the same concentration, with *G. biloba* always performing better than the *Z. officinale* rhizome at the same concentration and polarity. The order of *G. biloba* extract from superior to inferior was ethanol, ethyl acetate, n-butanol, chloroform, water, and petroleum ether (except that the petroleum ether part performed slightly better than the water part at 0.4 and 0.5 mg/ml), while the order of *Z. officinale* rhizome extract from superior to inferior was ethanol, chloroform, n-butanol, ethyl acetate, water, and petroleum ether. These two plant extracts demonstrated good *in vitro* effect against oxygen free radicals; the scavenging rate of superoxide free radicals had a significant positive correlation with the cell survival rate. The *in vivo* central nerve cell activity and SOD, glutathione peroxidase (GSH-PX) activity in aged mice blood serum increased while the MDA content decreased with treatment with these two extracts (*p* < 0.05). There were no significant changes in the number of leukocytes, lymphocytes, red blood cells, hemoglobin content, blood urine nitrogen, uric acid, creatinine, and the enzyme activity of glutamic oxaloacetic transaminase (GOT) and glutamic pyruvic transaminase (GPT) (*p* > 0.05). *G. biloba* had a better *in vivo* effect than *Z. officinale* rhizome even though their concentration and polarity part were same. These results could provide some references for better development of these two plant extracts from Yongzhou in the field of neuroprotection.

## 1 Introduction

Aging and central nervous system diseases all exhibit pathological processes such as neuron injury and cell apoptosis, so improving and repairing neuronal injury is an important strategy for preventing aging and treating central nervous system diseases (Zhou et al., 2010). Plant extracts are playing an increasingly important role in neuroprotection and neurological disease treatment. Many plant extracts such as glossy ganoderma, pine bark, and purslane have been found to have excellent neuroprotective bioactivity—free radical scavenging and oxidative stress alleviation are considered important reasons for the neuroprotective functions of these plant extracts (Lu et al., 2014; Guo et al., 2016; Li, 2018).

*Ginkgo biloba* and *Zingiber officinale* are two dominant plant resources in Yongzhou, Hunan Province. Extract of *G. biloba* leaf is widely used in treatment of cardiovascular and cerebrovascular diseases for containing flavonoids, esters, and other active components. Research in recent years has found that it also demonstrates activities of free radical scavenging, cerebral blood flow improvement, apoptosis process inhibition, and cell survival rate improvement for injured neurons, and shows significant potential for development in treating Alzheimer’s disease and acute cerebral infarction (Rojas et al., 2012; Liu et al., 2016; Vellas et al., 2012; El-Borm et al., 2021). *Z. officinale* rhizome also contains volatile oil, gingerol, and other active components; it has a series of medicinal and health care functions—antibacterial, antioxidant, anti-aging, and decreasing blood pressure—and has a protective effect on central neurons injured by cerebral ischemia and ischemia-reperfusion. It significantly improved the learning and memory ability of Alzheimer’s disease model rats (Zeng et al., 2013; Pannangrong et al., 2020).

Climate and environment factors affect the composition and content of the active components in plants organs, and the activity of different polarity parts of a certain plant extract may be different. Therefore, comparing the activity of different polarity parts of plant extracts from specific producing areas and analyzing their physiological impact could provide a basis for precise clarification of medication and pharmacological mechanisms (Luo et al., 2018; Xu et al., 2018; Ren et al., 2017). Until now, there have been few reports on neuroprotective and antioxidant activity and their correlation between the two kinds of bioactivity regarding the extracts of *G. biloba* leaf and *Z. officinale* rhizome from Yongzhou, as well as whether their activities are polarity part and concentration dependent. Therefore, this study focused on these issues in order to provide some references for the development of these two plant extracts in the field of neuroprotection.

## 2 Materials and methods

### 2.1 Materials

*G. biloba* leaves were collected in Yongzhou base in August 2019, and fresh *Z. officinale* rhizomes were purchased from the local market. These leaves and rhizomes were brought back to laboratory in an ice box, dried to constant weight at 60°C after washing, then powdered and screened at 80 mesh. The two kinds of plant were identified by Dr. Huang Guowen in the School of Chemistry and Bioengineering.

### 2.2 Main instruments

Ultraviolet visible spectrophotometer (Shanghai spectrum instrument Co., Ltd., SP-756P); cell incubator (Shengke Instrument Equipment Co., Ltd.); microplate reader (Type 1510, Thermofisher); high-speed tissue grinder (KZ-II, Wuhan Servicebio Co., Ltd.); automatic biochemical analyzer (Chemray 800, Redu Life Technology Co., Ltd.); automatic blood cell analyzer (BC-2800vet, Mindray Medical Instrument Co., Ltd.).

### 2.3 Main reagent

N-methyl-D-aspartic acid (NMDA, Shanghai Yuanye Biology company); DMEM medium (Sigma-Aldrich company); trypsin (Amresco company); D-Hank’s solution (Sigma-Aldrich company); cytarabine (Ara-C, Sigma-Aldrich company); CCK-8 Kit (Shanghai Bogu Biotechnology Co., Ltd.); malondialdehyde kit (Nanjing Jiancheng Bioengineering Research Institute); superoxide dismutase kit (SOD, Nanjing Jiancheng Biology); GSH-PH Kit (Nanjing Jiancheng Bioengineering Institute); DMEM/F-12 medium (Hyclone company); PBS solution (1x, Solarbio company); trypan blue (Solarbio company); collagenase I (Solarbio company); cytarabine (Solarbio company); L-glutamine (Solarbio company).

### 2.4 Methods

#### 2.4.1 Extraction

Powder of *G. biloba* leaf or *Z. officinale* rhizome was put into ten beakers (100 g each beaker), 500 ml 75% ethanol was added, then continuously treated in a 400 W microwave for 5 min.0 Stand for 24 h, suction filtration, concentrate with reduced pressure to get the extraction paste, then bake it to a constant weight at 60°C.

#### 2.4.2 Preparation of different polarity part of the extracts

Dry the extract to constant weight and dissolve in an appropriate amount of distilled water to make a suspension. Five different polar reagents—petroleum ether, chloroform, ethyl acetate, n-butanol, and ethanol—are successively added to the suspension at a 1:1 volume ratio. Repeat three times for each reagent and then add the next reagent to obtain the correspondingly different polarity parts of petroleum ether, chloroform, ethyl acetate, n-butanol, and ethanol; the remaining part after all the above organic solvents extraction is the water part. Each extraction solution is evaporated and dried in water bath, then baked to a constant weight at 60°C.

#### 2.4.3 Preparation and *in vitro* neuronal cells culture

The thalamus of mice born less than 24 h was separated under sterile conditions with the aid of anatomical microscope, cut into about 0.5 mm^3^, washed twice with D-Hank’s solution, digested in 0.125% trypsin, incubated at 37°C for 10 min; add the culture medium containing 10% fetal bovine serum to terminate trypsin digestion, centrifuge to remove the supernatant, and then add the buffer and gently blow to make the cell suspension. Dilute the cell suspension with DMEM culture medium to the appropriate concentration, then inoculate into the culture plate, place it in the CO_2_ incubator at 37°C, replace the solution in half amount after 24 h and add 10 μMol/L Ara-C to inhibit glial cells. Replace whole solution 48 h later, and then half solution replaced every three days; observe cell growth dynamically. Newborn mice are produced by adult mice fed in the laboratory which were purchased from Hunan Silaike Jingda Laboratory Animal Co., Ltd. Animal experiments were carried out after being approved by the ethics committee of the college.

#### 2.4.4 *In vitro* neuron injury and neuroprotective activity and antioxidant activity test

The cell suspension was inoculated into 96 well culture plates at 0.2 ml per well after five days’ culture. Except for a part of the cell culture wells left as the blank control group without NMDA added, NMDA is added to other culture wells at 50 μ Mol/L final concentration for induced neuronal injury. After NMDA treatment for the hypoxia injury group (no extract added), different concentrations of extract treatment groups of *G. biloba* leaf and *Z. officinale* rhizome in each polarity part were set. Added to each treatment group 0.2 ml were different concentration extracts, with 0.2 ml of 1% sodium carboxymethyl cellulose added to the blank control group and hypoxia injury group. The cell survival rate (CCK8 method), malondialdehyde (MDA) content, and superoxide dismutase (SOD) activity were then tested 24 h later (Xu et al., 2015; Yuan et al., 2016).

Take Tris-HCl buffer 8.0 ml (PH8.2) in a water bath at 25°C for 20 min, add 1.0 ml extract solution of each polarity part with different concentrations, and 0.5 ml 25 nmol/L pyrogallol hydrochloric acid solution preheated to 25°C; shake quickly, terminate reaction accurately with 0.5 ml concentrated HCl 4 min later, then determine the absorption value A_1_ at 320 nm. The absorption value (A_0_) was measured following the same steps except for replacing the extract solution with the same volume of distilled water; the absorption value A_2_ was measured following the some steps except for replacing pyrogallol hydrochloric acid solution with the same volume of distilled water. Each determination was repeated thrice to calculate the average value. The O_2_^−·^ scavenging capacity (%) of the extract solution can then be calculated with the formula [A_0_ − (A_1_ − A_2_)] * 100/A_0_ (Kong et al., 2015).

#### 2.4.5 Effect of the best polarity part on blood physiology and neuron cell activity of aged mice

Dissolve 0.2, 0.4 and 0.8 g of the best polarity part determined by *in vitro* experiment of the two extracts with normal saline and fix the volume to 100 ml to achieve three different concentration levels of 2 mg/ml (low), 4 mg/ml (medium), and 8 mg/ml (high). A total of 70 aged mice (20 months) were randomly divided into seven groups (ten mice each group) after five days adaptive feeding. The seven groups, including control group, were treated in groups with low, medium, and high concentrations of *G. biloba* and *Z. officinale* extract. Each mouse was fed with same amount every day, maintained a 25°C feeding environment temperature and 60% relative humidity. Extract solutions of 5 ml with low, medium, and high concentration were gavaged at 9 a.m. every day. The control group was fed with the same amount of normal saline. On the 20th day of gavage treatment, blood was collected from the tail after anesthesia, and blood physiological and biochemical indexes were determined by automatic biochemical analyzer and automatic blood cell analyzer (Chen et al., 2019). MDA content, SOD, and GSH-Px activity were determined according to the kit instructions.

After anesthesia and blood collection, the necks were broken and brain tissue was extracted and immediately immersed in the precooled DMEM/F_12_ serum-free medium. The hippocampus was then separated into the PBS buffer under an ice bath on an ultra-clean workbench. After washing with PBS five times, cut the hippocampus into small blocks, and then digest with 0.25% trypsin + 0.1% type I collagenase at 37°C for 40 min. Terminate digestion with FBS, mix gently, filter with 100 μm membrane, centrifuge the filtrate at 1,500 rpm for 5 min to remove the supernatant, then re-suspend with the complete medium (89% DMEM/F_12_ + 10% fetal bovine serum + 1% cyanine/streptomycin) and culture in an incubator. Update the solution by a half amount every two days, then add 8 μ cytarabine three days later to inhibit the proliferation of non-neural cells and primary glia. After that, add 10 μl CCK-8 solution per well on the fifth day, determine absorption value at 450 nm with the microplate reader after 2 h incubation, and then calculate the cell activity (%) (Wang et al., 2021).

## 3 Results and analysis

### 3.1 *In vitro* neuroprotective activity of different polarity parts of two kinds of extract

**FIGURE 1:**
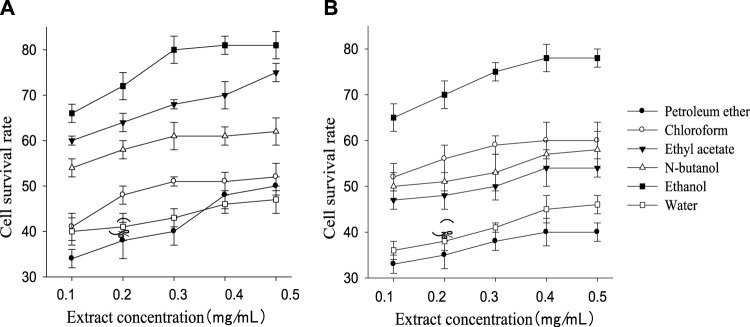
Survival rate of nerve cells in different polarity parts of extract of *G. biloba* leaf **(A)** and *Z. officinale* rhizome **(B)** at different concentration.

The cell survival rate of the treatment groups was higher than the injury group (32%) although still lower than the control group (94%). The cell survival rate of each treatment group increased with the rising extract concentration within a certain range and then kept stable (Figure 1). The order of the cell survival rate of the different polarity part of the extract of *G. biloba* leaf from high to low was ethanol, ethyl acetate, n-butanol, chloroform, water, and petroleum ether (petroleum ether part performed slightly better than water part at 0.4 mg/ml and 0.5 mg/ml), while the order of *Z. officinale* extract was ethanol, chloroform, n-butanol, ethyl acetate, water, and petroleum ether. Both kinds of extract performed best in ethanol part, and *G. biloba* always performed better than *Z. officinale* in all polarity parts except the chloroform part.

### 3.2 *In vitro* effect of the two extracts on the SOD activity and MDA content of the neuron cells

**FIGURE 2:**
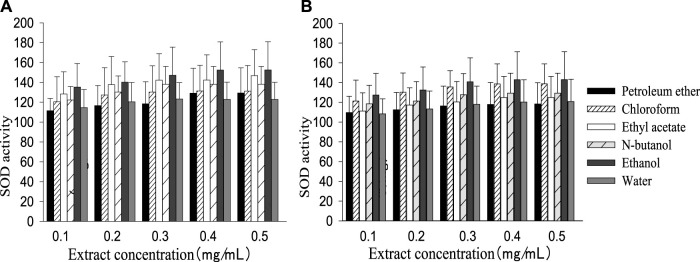
SOD activity in different polarity parts of the two plant extracts with different concentrations [**(A)**: *G. biloba* leaf; **(B)** *Z. officinale* rhizome].

**FIGURE 3:**
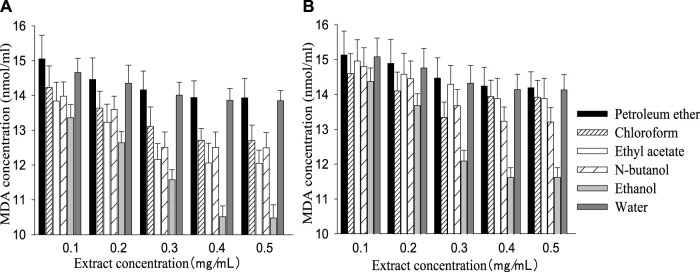
MDA concentration in different polarity parts of the two extracts with different concentrations [**(A)**: *G. biloba* leaf; **(B)** *Z. officinale* rhizome].

The SOD activity of the control group (161.20 ± 30.34) was higher than the injury group (103.36 ± 24.12), while MDA content (9.76 ± 0.35) was lower than the injury group (15.23 ± 1.16). Like the cell survival rate, SOD activity and MDA content increased or decreased with a rising extract concentration, then remained stable when the concentration reached a certain value (Figures 2, 3). The best performance concentration in ethanol part of the two extracts was different from other polarity parts, while all different polarity parts showed a similar overall law of change with increasing concentration. Both kinds of extract performed best in the polarity part of ethanol, and the performed order from superior to inferior of different polarity parts was consistent with the cell survival rate.

### 3.3 *In vitro* antioxidant activity of different polarity parts of the two extracts

**FIGURE 4:**
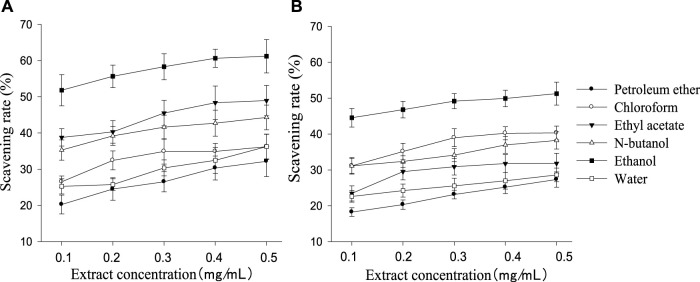
Scavening rate to superoxide radical in different polarity parts of the extracts with different concentrations [**(A)**: *G. biloba* leaf; **(B)** *Z. officinale* rhizome].

**FIGURE 5:**
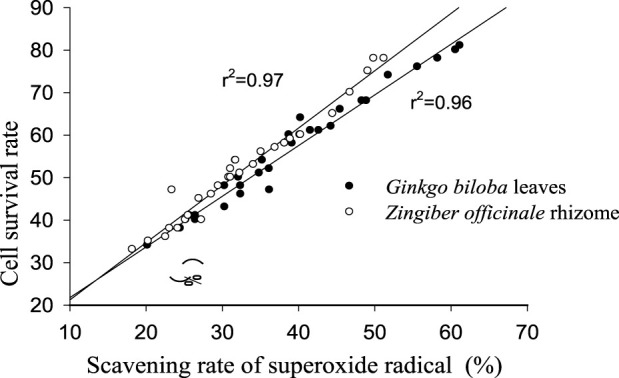
Correlation between cell survival rate and superoxide radical scavenging rate under treatment by the two plant extracts.

All polarity parts of the two plant extracts displayed different antagonistic effects on superoxide free radicals. The scavenging rate to free radicals increased with the rise in extract concentration in a certain range, and the order of different polar segments performing from superior to inferior was also consistent with the resulting cell survival rate (Figure 4). *G. biloba* performed better than *Z. officinale* if they had a same concentration and polarity part. As shown in Figure 5, there was a significant positive relationship between cell survival rate and superoxide radical scavenging rate under treatment with both *G. biloba* and *Z. officinale* extracts (*p* < 0.01).

### 3.4 *In vivo* effect of the best polarity part of the two extracts on the central neuron cell activity and blood physiology of aged mice

**FIGURE 6:**
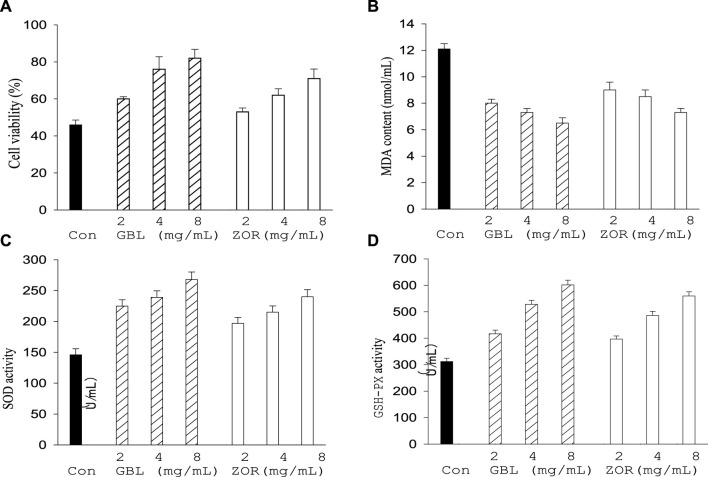
Effect of *G. biloba* leaf (GBL) and *Z. officinale* rhizome extracts (ZOR) from Yongzhou on central neuron cell viability **(A)** and serum antioxidant indexes **(B–D)** in aged mice.

After continuous gavage with ethanol polarity part of extracts of *G. biloba* leaf or *Z. officinale* rhizome, the activity of thalamic neurons (Figure 6A), SOD (Figure 6C), and GSH-Px (Figure 6D) in the serum of aged mice were higher than the control group (*p* < 0.05), while the MDA content decreased compared with the control group (Figure 6B). *G. biloba* performed better than *Z. officinale* when they had the same concentration level.

There were no significant differences in the number of white blood cells (WBC), lymphocytes, red blood cells (RBC), and hemoglobin content between the control and treatment groups (*p* > 0.05, Table 1), as well as the activities of glutamic oxaloacetic transaminase (GOT), glutamic pyruvic transaminase (GPT), and the contents of blood urine nitrogen (BUN), uric acid (UA), and creatinine (CEEA), which were closely related to liver and renal functioning (*p* > 0.05, Table 1).

**TABLE 1:** Effects of extracts of *G. biloba* leaves (GBL) and *Z. officinale* rhizome (ZOR) on aged mice’ blood physiology.

Indexes	GBL (mg/ml)			ZOR (mg/ml)			Con
	8	4	2	8	4	2	
WBC (10^9^/L)	10.0	10.3	10.6	10.4	10.4	10.2	10.3
Lymphocyte (10^9^/L)	6.6	6.7	6.8	6.9	6.8	6.8	6.8
RBC (10^9^/L)	7.2	7.2	7.2	7.2	7.1	7.1	7.2
Hemoglobin (10^12^/L)	142	140	140	140	140	141	140
GOT (U/L)	47.6	47.1	46.5	47.3	47.3	46.8	46.8
GPT (U/L)	167.8	160.4	161.8	165.8	165.6	161.7	166.5
BUN (mmol/L)	8.6	8.4	8.3	8.3	8.3	8.2	8.2
UA (μmol/L)	2.7	2.6	2.6	2.6	2.6	2.5	2.5
CREA (μmol/L)	75.6	74.1	74.5	75.1	73.6	72.8	72.3

## 3 Discussion

Neurodegenerative diseases such as Parkinson’s and Alzheimer’s diseases are closely related to neuron damage in specific central neuron parts, and clinical symptoms will appear when the number of injured neuron cells reaches a certain proportion (dopamine neuron numbers in the substantia nigra of Alzheimer’s disease patients reduces more than half). It is, therefore, of great importance to screen the natural active ingredients with neuroprotective effects to prevent neurodegenerative processes and treat corresponding diseases. Local dominant and characteristic plant resources are important sources for active substance screening; these natural active substances with neuroprotective activity are mainly concentrated in glycosides, flavonoids, saponins, and so on (He et al., 2017). *G. biloba* leaf contains flavonoids and esters, while *Z. officinale* rhizomes contain curcumin, flavonoids, polyphenols, saponins, These two plant resource thus have the substance bases of neuroprotective function (Wang et al., 2011; Shang et al., 2018; Small et al., 2018).

This study found that all the polarity parts of the extracts of *G. biloba* leaf and *Z. officinale* rhizomes produced in Yongzhou had the effect of increasing the cell survival rate and SOD activity of central nerve cells while reducing the MDA content, meaning that the two plant extracts performed positively in central neuron survival and showed excellent neuroprotective activity. However, the composition and content difference of these active components in each polarity part led to a different presentation of protective effect, so it is necessary to separate, purify, and identify the components of the high activity polarity part of the two plant extracts. Coincidentally, both the extracts show the best activity in ethanol polarity part—the non-toxicity, low boiling point, and easy removal of ethanol are beneficial for its large-scale extraction and application.

Antagonizing oxidative stress and oxidative damage are considered important reasons for the neuroprotective activity of plant extracts. If oxidation and antioxidation are out of balance in cells, the predomination of oxidation could lead to free radicals enrichment which would damage cell membrane structures and cause a series of adverse consequences, including metabolic disorder, abnormal protein expression and mitochondrial function, and the failure of physiological activities (Rhein et al., 2010; Jia et al., 2011; Yan et al., 2014). In this study, the *in vitro* changes of SOD activity and MDA content with the concentration and scavenging ability to super-oxidize the free radicals of the two plant extracts indicate their excellent antioxidant activity. The positive correlation between cell survival rate and free radical clear rate indicate that antioxidant activity is an important underlying mechanism for the neuroprotective function of the two plant extracts.

Oxidative stress is considered an important cause of aging, while effective anti-oxidation can delay the aging process. *G. biloba* extract can inhibit the intracellular oxidative stress level of central neurons; *Z. officinale* extract can reduce lipid peroxidation in the brain tissue of Alzheimer’s model rats (Xia et al., 2014; Song et al., 2015). The ethanol polarity part improves body antioxidant function by affecting the MDA content and the activity of SOD, GSH-Px in blood serum. This indicates that the two plant extracts have neuroprotective efficacy and can slow the central nervous degeneration process by improving elderly individuals’ antioxidant capacity. There is no significant change in the aged mice blood cells numbers, the content of hemoglobin, uric acid and creatinine, and the activities of GOT and GPT in serum (*p* > 0.05). This indicates that the two extracts are very safe and will not cause a stress response to or damage the body’s immune system and the organ functions of the liver and kidney. *G. biloba* always performs better than *Z. officinale* when they have same concentration and polarity part; the activity of the two plant extracts shows an obvious concentration dependence within a certain range, providing important references for future neuroprotection formulation development based on these two plant extracts from Yongzhou (Rojas et al., 2012; Xin et al., 2015; Ren et al., 2017).

## Data Availability

The original contributions presented in the study are included in the article/supplementary material, further inquiries can be directed to the corresponding authors.
